# Pharmacokinetic Study of Biotransformation Products from an Anxiolytic Fraction of *Tilia americana*

**DOI:** 10.3390/molecules22081260

**Published:** 2017-07-27

**Authors:** Virgilio Alfonso Juárez Ramírez, María Isabel Jiménez-Beltrán, Alejandro Zamilpa, Maribel Herrera-Ruiz, Rodolfo Abarca-Vargas, Galia Lombardo-Earl, Jaime Tortoriello, Enrique Jiménez-Ferrer

**Affiliations:** 1 Centro de Investigación Biomédica del Sur, Instituto Mexicano del Seguro Social (IMSS), Argentina No. 1, CP 62790 Xochitepec, Morelos, Mexico; virgis69@hotmail.com (V.A.J.R.); jbmi_ff@uaem.mx (M.I.J.-B.); azamilpa_2000@yahoo.com.mx (A.Z.); cibis_herj@yahoo.com.mx (M.H.-R.); rodolfo.abarcav@outlook.com (R.A.-V.); galia100000@yahoo.com.mx (G.L.-E.); jtortora2@yahoo.com.es (J.T.); 2 Doctorado en Farmacia, Facultad de Farmacia. Universidad Autónoma del Estado de Morelos, Av. Universidad No. 1001, CP 62209 Cuernavaca, Morelos, Mexico

**Keywords:** *Tilia americana*, anxiolytic, pharmacokinetic

## Abstract

An anxiolytic fraction of *Tilia americana* standardized in tiliroside, rutin, quercitrin, quercetin glucoside, and kaempferol was obtained. After oral administration of the fraction, the above-mentioned flavonoids were not detected in plasma over 24 h. However, *meta* and *para* hydroxyphenylacetic acid and dihydroxyphenylacetic acid (*m*-HPAA, *p*-HPAA and DOPAC) were monitored. These are the biotransformation compounds of the aglycones of kaempferol and quercetin; these aglycones are products of the other flavonoids present in the anxiolytic fraction. The analytical methods (HPLC) for flavonoids and the related compounds (*m*-HPAA, *p*-HPAA and DOPAC) were validated, determining the parameters of accuracy, precision, specificity or selectivity, limit of detection, quantification range, and robustness. The pharmacokinetic assay was performed with ICR mice strains, which were given 200 mg/kg of the standardized active fraction. The results of validation of the analytical methods were obtained, allowing it to be established in a validated way that no flavonoids present in the anxiolytic fraction of *T. americana* were detected in plasma. However, detection and follow up was possible for the serum levels of *m*-HPAA, *p*-HPAA, and DOPAC. The three compounds follow a two-compartment model with very similar parameters between *m*-HPAA and *p*-HPAA, some being different from the ones characterized in the pharmacokinetics of DOPAC.

## 1. Introduction

Anxiety disorders have a current prevalence of 2.4 to 29.8% of the population in the world [1]. The main expression of the generalized anxiety disorder (GAD) has treatments that use drugs like selective reuptake inhibitors (SSRIs) or norepinephrine/noradrenaline selective reuptake inhibitors (SNRI), as well as benzodiazepines (BZD), which are the second most used pharmacological treatment [2]. While the first two are effective anxiolytics, they require a long period of administration to present the desired pharmacological effects, a period which can exacerbate the symptoms of anxiety. BZD administration causes rapid pharmacological effects to control the symptoms of GAD. However, BZD should be used for a short time only since they cause different side effects such as drowsiness, falls, confusion, impairment of memory, and incoordination (which can be particularly problematic in the elderly). Also, BZD generates tolerance and a tendency for dependence and potentially a substance abuse [2].

In Mexican traditional medicine, *Tilia americana* is used for the treatment of nervous disorders; the fresh inflorescence is used to conciliate sleep and to reduce the hangover sensations from insomnia, which usually is accompanied by headaches and poor general condition [3,4]. The above has been proven experimentally with pharmacological studies in murine models [5,6,7].

**Figure 1:**
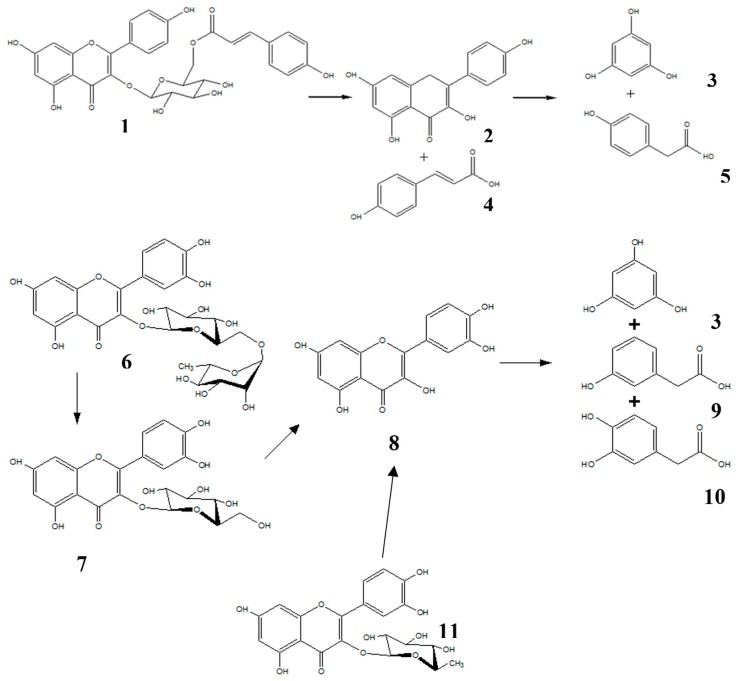
Flavonoid biotransformation process of tilliroside (**1**), rutin (**6**), quercetin glucoside (**7**), kaempferol (**2**), quercetin glucoside (**8**) and products obtained; phloroglucinol (**3**), coumaric acid (**4**), para-hidroxyphenyl acetic acid (*p*-HPAA) (**5**), 3,4-dihydrophenylacetic acid (DOPAC) (**10**), meta-hydroxiphenyl acetic acid (*m*-HPAA) (**9**), and quercitrin (**11**).

Therefore, our research group established the anxiolytic effect of the aerial parts of *Tilia americana* var. mexicana (Schltdl) Hardin (Tiliaceae) [7]. Consequently, it was possible to determine the composition (mg/g of extract) of an active fraction, characterized by the presence of five flavonoids; tiliroside 28.56 (**1**), quercitrin 7.96 (**11**), rutin 3.93 (**6**), quercetin glucoside 16.25 (**7**), and kaempherol 2.83 (**2**) [7,8]. The initial purpose of the study was to determine the plasma concentration of the flavonoids present in the anxiolytic fraction from *T. americana*. However, this could not be done since the plasma of the mice that were administered orally with said fraction did not present any of these glycosylated compounds or their corresponding aglycones. Therefore, the aim of this work was redirected, and monitoring the variation of plasma concentration of the products that were obtained from the degradation of the flavonoids present in the anxiolytic fraction was carried out. Figure 1 shows the hydrolysis reaction scheme of the flavonoids of the active fraction; the products obtained were para-hydroxyphenylacetic acid (*p*-HPAA) (**5**)**,** meta-hydroxyphenylacetic acid (*m*-HPAA) (**9**), and 3,4-dihydroxyphenylacetic acid (DOPAC) (**10**). Previous studies of these compounds have shown a clear anxiolytic effect [9].

## 2. Results and Discussion

### 2.1. Chromatographic Profiling of Flavonoids, m-HPAA, p-HPAA and DOPAC Standards

**Figure 2:**
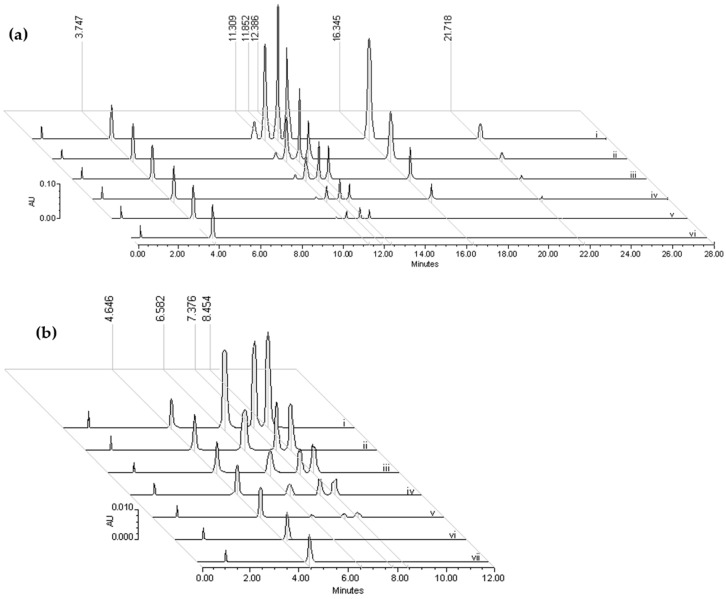
(**a**) Chromatographic profile of the concentration curves of the flavonoids (standards) present in the anxiolytic fraction of *T. Americana*; and (**b**) the concentration curves of standards of *m*-HPAA, *p*-HPAA and DOPAC.

Figure 2 shows the results of the calibration curves for the flavonoids present in the anxiolytic fraction of *T. Americana*, shown in Figure 2a, and the compounds generated from the biotransformation of the aglycones kaempferol and quercetin, derived from the three flavonoids. The anxiolytic fraction was obtained from the *T. americana* extract, and the compounds *m*-HPAA, *p*-HPAA, and DOPAC were a mix prepared from commercially acquired chemical standards. In Figure 2a, the chromatograms from i to vi are presented from the highest to lowest concentration of flavonoids. These include rutin (RT = 11.309 min; 5.4, 2.7, 1.35, 0.67 and 0.34 µg/mL); quercetin glucoside (RT = 11.852 min); quercitrin (RT = 12.386 min); tiliroside (RT = 16.345 min) and each substance with the same pattern dilution concentrations (56.8, 28.4, 14.2, 7.1 and 3.6 µg/mL); and kaempferol (RT = 21.718 min; 8.9, 4.4, 2.2, 1.1 and 0.5 µg/mL). The internal standard used was acetaminophen (RT = 3.747 min, 2.0 µg/mL).

Regarding Figure 2b, the chromatograms of the compounds resulting from the biotransformation of kaempferol and rutin are presented, which are the two aglycones derived from the flavonoids of the active fraction of *T. americana*. Similarly, the profiles were ordered from the highest to the lowest concentration and are identified from i to vii. They include *m*-HPAA (RT = 6.582 min), *p*-HPAA (RT = 7.376 min), DOPAC (RT = 8.454 min), and each substance with the same pattern dilution concentrations (31.2, 15.6, 7.8, 3.9, 1.9 and 0.97 µg/mL). The internal standard used was dopamine (RT = 4.646 min, 2.0 µg/mL). Chromatograms vi in Figure 2a and vii in Figure 2b, correspond to the plasma samples without the flavonoids or the biotransformation products added, respectively.

### 2.2. Validation Methods

#### 2.2.1. Standardization of the Chromatographic Process

The methods were validated using the following criteria; for linearity and sensitivity, the data of the flavonoids were found to be linear over a concentration range of 0.34 to 54.6 μg/mL, and the products of the biotransformation of the flavonoids were found to be linear over a concentration range of 0.97 to 31.5 μg/mL. The regression equation for rutin was (y) = 0.0917 (x) + 0.0103; r^2^ = 0.989; quercetin glucoside showed the regression equation (y) = 0.0117 (x) + 0.0445; r^2^ = 0.988. The equation of the fitted line of quercitrin was (y) = 0.0207 (x) + 0.0717; r^2^ = 0.988. For tiliroside, the result of the linear regression was (y) = 0.0744 (x) + 0.058; r^2^ = 0.996. For kaempferol, the linear regression of the standard curve was (y) = 0.0091 (x) + 0.0062; r^2^ = 0.992. Regarding the compounds from the biotransformation of the flavonoids, the linear regression calculations of the standard curves were *m*-HPAA (y) = 0.091 (x) + 0.0098; r^2^ = 0.996. For *p*-HPAA, (y) = 0.073 (x) + 0.0456; r^2^ = 0.997, and, for DOPAC, (y) = 0.104 (x) + 0.0091; r^2^ = 0.996. If r > 0.98, this indicates good linearity. The limits of detection (LOD) determined were for rutin 0.17 µg/mL, quercetin glucoside 1.27 µg/mL, quercitrin 1.27 µg/mL, tiliroside 1.24 µg/mL, kaempferol 0.28 µg/mL, *m*-HPAA 0.06 µg/mL, *p*-HPAA 0.14 µg/mL, and DOPAC = 0.5 µg/mL, with a value of precision relative standard deviation (RSD) < 20%. Regarding the values of the limit of quantification (LOQ), these were for rutin 0.53 µg/mL, quercetin glucoside 3.85 µg/mL, quercitrin 3.84 µg/mL, tiliroside 3.77 µg/mL, kaempferol 0.86 µg/mL, *m*-HPAA 0.20 µg/mL, *p*-HPAA 0.43 µg/mL, and DOPAC = 0.15 µg/mL, with a value of precision (RSD) < 20% (Table S1). Under these chromatographic conditions, the number of theoretical plates of the column was between 26700 < N > 18760 for flavonoid type compounds. The parameter for the biotransformation products of the flavonoids was between 4324 < N > 1706, which was acceptable in both cases in terms of separation efficiency. The resolution of the separation [Rs] system was calculated for the adjacent peaks; the results indicated that the Rs values were in the range of 19.42 < Rs > 1.5 for tiliroside versus kaempferol and quercetin glucoside versus quercitrin, respectively. The biotransformation products system from the flavonoids showed Rs values of 1.8 and 2.5 for *p*-HPAA versus *m*-HPAA and DOPAC versus *p*-HPAA, respectively. In any case, the system resolution was acceptable since Rs > 1.5.

No interference was observed between any plasma constituents with the anxiolytic *T. americana* active fraction rich in flavonoids or with the biotransformation products thereof. Flavonoids and internal standards were ruled out by analyzing the chromatograms of the blank plasma. (Figure 2, sample vi Figure 2a and sample vii Figure 2b). 

Regarding the specificity of the procedure, acetaminophen was used as an internal standard for the determination of flavonoids and was eluted at minute 3.74, and the first flavonoid (rutin) of the mixture was eluted at minute 11.31 (Figure 2a). Furthermore, regarding the flavonoids’ biotransformation products, dopamine, which was the internal standard, was eluted at minute 4.64, and *m*-HPAA was eluted at minute 6.58 (Figure 2b). In both cases, the internal standards present a good separation in elution time regarding the first two analytical systems (Figure 2). There is no disturbance from the background signals in the plasma after the extraction with methanol (Figure 2). 

The intra and inter-assay accuracies (Table S2) were expressed as the percent differences between the measured concentration and nominal concentration. Intra-assay precision and accuracy were calculated using replicas (*n* = 6) for the determination for each concentration of the spiked plasma sample during a single analytical run. Inter-assay precision and accuracy were calculated using replicate (*n* = 6) determinations of each concentration made on three separate days. Accuracy (% Bias) = [(Cobs − Cnom)/Cnom] × 100. The precision (relative standard deviation; RSD) was calculated from the observed concentrations as follows; RSD = [standard deviation (SD)/Cobs] × 100. Accuracy (Bias) and precision (RSD) values ± 15% covering the range of actual experimental concentration were considered acceptable [10].

#### 2.2.2. Standardization of the Extraction Process

The extraction efficiency (Table S3) of the five flavonoids present in the anxiolytic fraction and the biotransformation compounds of kaempferol and quercetin glucosides were determined by analyzing sets (*n* = 6). The concentrations of the compounds that were extracted from the matrix (plasma) were variable; with rutin being concentrated at 1.75, 3.50, and 7.00 μg/mL; quercetin glucoside, quercetrin, and tiliroside at 11.02, 22.18, and 44.3 μg/mL; kaempferol at 1.12, 2.24, and 4.50 μg/mL; and *m*-HPAA, *p-*HPAA, and DOPAC at 1.95, 7.84, and 31.25 μg/mL, representing low, medium, and high quality control (QCs), respectively. Recovery was calculated by comparing the peak areas of each flavonoid and of the compounds derived from the biotransformation of the flavonoids added into the blank samples.

The stability of the flavonoids present in the anxiolytic fraction (rutin, quercetin glucoside, quercetin, tiliroside, and kaempferol) and the compounds derived from the biotransformation of kaempferol and quercetin glucoside are presented in Table 1. The precision of the samples kept at room temperature (Table 1) was maintained in a range between 0.09 to 9.99%, and the accuracy ranged from −8.12 to 7.83%. The results indicate that flavonoids and related compounds were stable when stored at −70 °C for at least one month; the precision varied between 0.27 to 10.02% and the accuracy from −3.52 to 7.26%. The precision for the autosampler stability ranged from 0.06 to 10.58%, and the accuracy ranged from −7.77 to 5.48%.

These results suggest that the flavonoids present in the anxiolytic fraction and the related compounds could be adequately analyzed due to the stability of the samples in the storage processes to which the actual samples from experimental animals would be subjected.

### 2.3. Plasma Level of Flavonoids and Relative Products

The initial results of the variation in plasma concentration of the flavonoids (tiliroside, kaempferol, rutin, quecitrin, and quercetin glucoside) administered orally were unexpected since these were not found in the plasma immediately after administration (Table 2). Consequently, a follow-up of the pharmacokinetic process of the biotransformation products of these five flavonoids present in the active fraction was carried out, as reports discuss that the compounds derived from the biotransformation process of flavonoids that occur in the gastrointestinal tract are the ones responsible for the anxiolytic effect of said flavonoids [9]. On the other hand, some evidence mentions that flavonoids with anxiolytic activity found in other *Tilia* species do not present the same pharmacological effects when administered parenterally as when given orally [11]. This could be due to the absorption of flavonoids that can vary depending on the level of glycosylations; i.e., in an intestinal epithelial permeability study, the absorption of tiliroside compounds was less efficient for the glycosylated kaempherol, which was completely impermeable to the intestinal epithelial cells, compared with the aglycone kaempherol [12]. Likewise, rutin administered orally was not absorbed into the vascular space since the glucoronides and sulphates of quercetin obtained after the biotransformation process were found in (mice/human) plasma [13]. Flavonol hydroxylation that generates *m*-HPAA, *p*-HPAA, and DOCAP alters the processes of intestinal absorption [14,15]. Complex intestinal conversion results from the intervention of intestinal bacterial microflora and enterocytes making it difficult to predict the uptake of flavonoids and even their metabolites [16]. The flavonols kaempferol and quercetin were metabolized into floroglucinol that derives from the ring A of the flavonol; the differentiation of the biotransformation process is from the products obtained from ring B since kaempferol produces *p*-HPAA and quercetin generates DOPAC, which eventually becomes *p*-HPAA or *m*-HPAA [16].

As mentioned, the ansiolitic fraction of *T. americana* administerd contains five flavonoids, from which the aglicones that stand out are kaempferol y quercetin glucoside, as well as their biotransformation products; *m*-HPAA, *p*-HPAA, and DOPAC. Therefore, the following study was focused on these three products.

Table 2 shows the data obtained from the HPLC determination of the active fraction in plasma (kaempferol, rutin, quercetin glucoside, tiliroside, and quercitrin) and the compounds derived from the biotransformation of kaempferol and quecetin (*m*-HPAA, *p*-HPAA, and DOPAC). These determinations were performed at different times and are the mean of the quantification of the group of mice (*n* = 10).

The data shown in Table 2 were applied to the program PKSolver [17]. After a single oral administration of 200 mg/kg of the anxiolitic fraction of *T. americana*, the behavior of the concentration-time data allowed us to determine the main pharmacokinetic parameters under a two-compartment model (Table 3), and the same program provided the graphical behavior of the variables and the adjustment to the two-compartment model.

**Figure 3:**
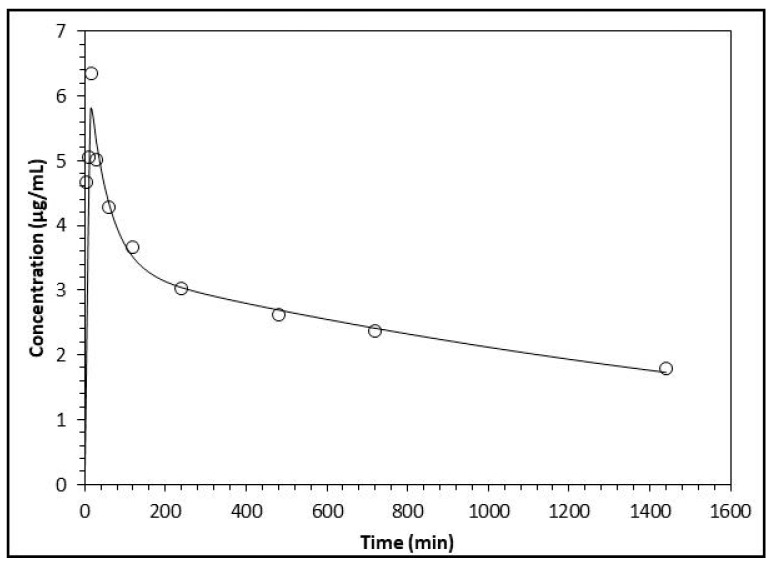
Analysis of the variation of plasma concentration of *m*-HPAA (open circles) with respect to the time in minutes. The line represents the prediction of the behavior of the concentration following a two-compartment model.

**Figure 4:**
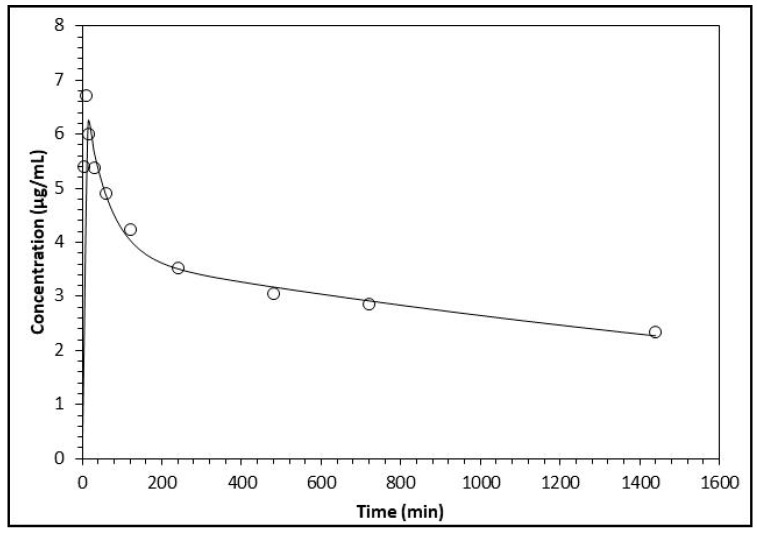
Analysis of the variation of plasma concentration of *p*-HPAA (open circles) with respect to the time in minutes. The line represents the prediction of the behavior of the concentration following a two-compartment model.

**Figure 5:**
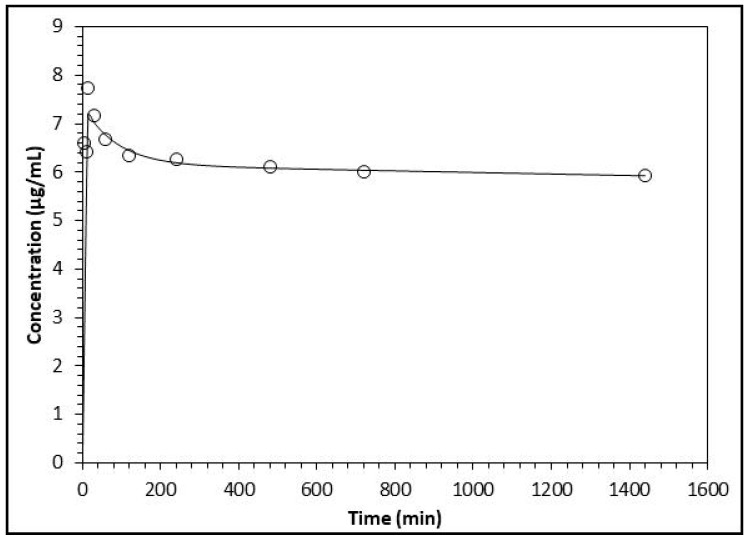
Analysis of the variation of plasma concentration of DOPAC (open circles) with respect to the time in minutes. The line represents the prediction of the behavior of the concentration following a two-compartment model.

The analysis of the pharmacokinetic behavior of the flavonoids’ biotransformation products made evident that the results match and are more suitable for the two-compartment model for all three compounds. This is indicated by the Akaike Information Criterion (AIC) and the Schwarz (SC) criterion. For the three biotransformation compounds, the values of AIC and SC are lower for the two-compartment model with respect to the one-compartment model (results not shown) [18]. Since the data fitted the two-compartment model, the pharmacokinetic parameters were analyzed by plotting the concentrations of the compounds over time; these fit better with the decay curve built by the software (Figure 3, Figure 4 and Figure 5).

Table 3 indicates that the concentration of DOPAC in the central compartment, compartment A, is half of the values reached by *m*-HPAA and *p*-HPAA. Contrary to what happens in the peripheral compartment, where the value of B was doubled. The apparent first-order absorption rate constant (ka) for *m*-HPAA showed the lowest magnitude, which was 24% and 79% smaller than the k_a_ of *p*-HPAA and DOPAC, correspondingly. The difference between the values of k_a_ for *m*-HPAA and DOPAC can explain the difference in the values of A and B. DOPAC reaches five times the concentration in the peripheral compartment than in the central compartment. As there is a low elimination rate, there is a reaction that favors the DOPAC in the peripheral compartment, as the drug elimination rate (k_10_) was thirty times lower for DOPAC than for *m*-HPAA, although the values of the return rate of the peripheral compartment to the central compartment (k_21_) were very similar. In this way, a substantially higher retention time t_1/2_ β was achieved for DOPAC in contrast with the residence time in the central compartment t_1/2_ α and was not so different when comparing the values reached by the three products. The values of the retention times were determined by the mass exchange between the central compartment and the peripheral compartment. The above comparison was defined by the relation between rate constants of k_12_ with respect to k_21_. It could be considered that *m*-HPAA was accumulated with a higher index of 0.9 with respect to *p*-HPAA, with 0.77, and to what could be expected for DOPAC, which had the lowest index of 0.20. The difference in both the k_10_ and the index values indicate DOPAC accumulation in the peripheral compartment; the above was measured by the Area Under the Curve (AUC_0→∞_ and ABC_0–1440_) and main residence time (MRT). In terms of these three parameters, the values obtained for DOPAC were higher than those reached by *m*-HPAA and *p*-HPAA. On the other hand, when the values of metabolite distribution such as Apparent Volume Distribution after non-intravenous administration (V/F) were compared, the values were very similar for the three drugs. Thus, in terms of the total apparent clearance of the plasma drug after oral administration (CL/F), the determined values for DOPAC were 30 and 20 times lower than those for *m*-HPAA and *p*-HPAA respectively.

## 3. Materials and Methods

### 3.1. Chemicals

The following chemicals were used: HPLC grade acetonitrile, methanol, and water (Merck, KGaA, Darmstadt, Germany); trifluoroacetic acid (Mallinckrodt Inc., Phillispburg, NJ, USA); orthophthalaldehyde, *n*-hexane, ethyl-acetate, and methanol (Merck, Naucalpan de Juárez, México state, Mexico); Rutin, Quercitrin, Kaempherol, Quercetin, para-hidroxyphenyl acetic acid, 3,4-dihydrophenylacetic acid, and meta-hydroxiphenyl acetic acid (*m-*HPAA) (Sigma-Aldrich, Saint Louis, MO, USA).

### 3.2. Plant Material

Aerial parts of *Tilia americana* var. mexicana (Schltdl) Hardin (Tiliaceae) were collected from a wooded area of Mexicapan, State of Mexico, Mexico (18°59′38.01″ N and 99°19′17.95″ W, 2,281 m.a.s.l.). The plant material was identified by Abigail Aguilar-Contreras, M.Sc. (Instituto Mexicano del Seguro Social [IMSS]M Herbarium, Mexico); specimens were stored at this site for future reference with voucher number (IMSSM-15099). The collected material was separated by organs and dried under dark conditions at room temperature for two weeks. Dry material was pulverized in an electric grinder (Pulvex), obtaining particles of <4 mm.

### 3.3. Extraction Process of Tilia americana

Plant material was extracted by maceration; three different solvents were used, starting with n-hexane, then ethyl acetate, followed with methanol for the extraction of the residual material. The whole procedure was carried out at room temperature (23 ± 2 °C). Each extract was dried by eliminating the solvents by reduced pressure distillation with a Heidolph-brand (Heidolph Intruments GmbH & Co.KG, Schwabach, Germany) rotatory evaporator.

#### Active Fraction Preparation

Based on the methodology proposed by Herrera-Ruiz et al., (2008) and Nogueron-Merino et al. (2015), the methanol extract was used for the fraction separation process; this extract was subjected to a liquid–liquid extraction with ethyl acetate and water (1:1, 500 mL). The aqueous fraction that was obtained was then re-extracted by a bipartition with dichloromethane, acetone, and methanol; each of these respectively removed compounds of interest from the aqueous fraction. Finally, these three fractions were mixed together, generating the anxiolytic fraction, which was then subjected to the pharmacokinetic characterization.

### 3.4. Animals

ICR albino mice of 30g to 36 g were used (Harlan México, D.F.). Animals were kept in groups of 8 to 10 animals per cage, maintained under laboratory conditions at 25 °C, with a 12 h light/dark schedule (lights were put on at 07:00 a.m.), and had ad libitum access to water and food (pellets from Harlan Rodent Lab Diet). The adaptation time to the laboratory conditions before experiments was three weeks. All the studies were implemented in accordance to the Mexican Official Regulation NOM-062-ZOO-1999 (Technical Specifications for Production, Care and Use of Laboratory Animals). The institution that authorizes the ethical use of animals approved the project through the local health research committee (IMSS), which was registered under the number R-2013-1701-30. To obtain consistent data, the minimum number of animals and time of observation were used.

### 3.5. Standards and Internal Standard Stock Solutions for Chromatographic Profiling

Flavonoid standards were prepared separately, by dissolving 50 mg of each of the following flavonoids in 50 mL of methanol; quercitrin, isoquercitrin, tiliroside, rutin, and kaempferol. Subsequently a mixture with different concentrations of these flavonoids was made, simulating the proportions found in the active fraction. A final volume of 1 mL was prepared with the following proportions; for quercitrin, isoquercitrin, and tiliroside 50 µg of each was used, with 9 µg of rutin and 5 µg of kaempferol.

The standards for *p*-HPAA, *m*-HPAA, and DOPAC (which are the flavonoids’ biotransformation products) were prepared separately by dissolving 50 mg of each one in 50 mL of methanol. A mixture was also done for the chromatographic analysis; the proportion was 50 µg of each compound in a final volume of 1 mL. 

Acetaminophen and Dopamine were used as the internal standard stock solutions (SI), which were prepared individually by dissolving 0.20 mg of each one in 100 mL of methanol (concentration of 2 µg/mL). These were added to the flavonoid mix and the biotransformation product mix, respectively.

### 3.6. HPLC Calibration Curve of Flavonoids, p-HPAA, m-HPAA and DOPAC in Plasma

Plasma samples were prepared with the flavonoids *p*-HPAA, *m*-HPAA, and DOPAC according to the standards presented above by adding the stock solutions to the mice plasma. The proportions of the concentration curve were as follows: for Quercetin glucoside, Quercitrin, and Tiliroside (54.6, 58.4, 14.1, 7.1 and 3.6, µg/mL); Rutin (5.4, 2.7, 1.35, 0.67 and 0.34 µg/mL); Kaempferol (8.9, 4.4, 2.2, 1.1 and 0.5 µg/mL); and for *m-*HPAA, *p-*HPAA, and DOPAC (31.2, 15.6, 7.8, 3.9, 1.9 and 0.97 µg/mL).

For the accuracy and precision determinations, quality control (QC) samples were used and were prepared individually at three concentrations (low, medium, and high) as follows (Table 4).

### 3.7. Experimental Design

Eleven groups of ten animals each were randomly formed. All animals were orally administered with 200 mg/kg of the active fraction. Animals were anaesthetized with ether, and blood samples were obtained from the retroorbital sinus with a heparinized capillary, each group was examined at different times (0, 5, 10, 15, 30, 60, 120, 240, 480, 720, and 1440 min) after the active fraction was administered. Each blood sample that was obtained had a volume of 700 to 1000 µL, which was collected in heparinized centrifuge microtubes and centrifuged for 5 min at 1720× *g*. Then the plasma samples were frozen and lyophilized. The dry plasma was subsequently used to extract and measure the concentration of the flavonoids and biotransformation products. 

### 3.8. Quantification of Flavonoids and Biotransformation Products in Plasma

Plasma dried pulverized samples were extracted with methanol (HPLC grade, Merck). The extracts were filtered with Teflon membrane dimensions 0.45 μm by 13 mm (Millex^®^-LCR); 20 μL of each filtered sample was injected into the HPLC equipment. The measurements were performed in a (Waters corporation, Milford, CT, USA) 2695 HPLC with a diode array detector (Waters 2996) and processed with the Empower Pro 1.0 software. A Chromolith Performance^®^ RP-18e 100 × 4.6 mm (Merck KGaA, Darmstadt, Germany) was used. The mobile phase consisted of a mixture of acetonitrile and water with the proportion 55:45 and a flow rate of 1 mL/min. The concentrations of the flavonoids and the biotransformation products were obtained by comparison with a pre-built calibration curve at λ = 220 nm.

### 3.9. HPLC Calibration Curve for Flavonoids and Biotransformation Products

Towards the determination of the concentration of the flavonoids and biotransformation products contained in the plasma samples, a calibration curve was developed with a HPLC system. In an initial solution of 1 mg/mL, the active fraction was dissolved in methanol (HPLC grade) and was then used to prepare successive dilutions to establish decreasing concentrations of the flavonoids and biotransformation products (62.5, 15.6, 3.9, 0.98, and 0.24 μg/mL). These were injected into the HPLC in triplicate.

### 3.10. Validation Method

Validation methods were performed according to the Food and Drug Administration (FDA) guidelines for analytical validation methods.

### 3.11. Linearity and Sensitivity Test

For the evaluation of the linearity of the standard calibration curves, determinations of the flavonoids and the biotransformation products in plasma samples were accomplished on three independent days using freshly prepared samples. 

Calibration curves for the plasma samples were prepared throughout a linear range of 0.24 to 62.5 μg/mL. Each calibration curve was compared against a double blank sample with and without internal standards and five calibration concentrations.

Each calibration curve was constructed by plotting the component (analyte) to an internal standard peak area ratio (y) against the analyte concentrations (x). The calibration curves were fitted using a linear least square regression model (y) = m(x) + b using Microsoft Office Excel 2010 software. The resulting m and b parameters were used to determine the back-calculated concentrations that were evaluated statistically. All calibration curves of the flavonoids and the biotransformation products were created before the experiments with linear correlation values of at least 0.9995.

### 3.12. Specificity Test

The specificity test was defined by two conditions; a non-interference term when the flavonoids and the biotransformation products were not retained by the endogenous components of plasma and, secondly, no cross-interference between the flavonoids or the biotransformation products with the internal standard using the proposed extraction procedure and HPLC conditions. Six different plasma samples were used as blanks (of the flavonoids and the biotransformation products free plasma) and were evaluated with and without internal standard to assess the specificity of the method.

### 3.13. Accuracy and Precision Test

The intra- and inter-assay accuracies were expressed as the percentage difference between the measured concentration and the nominal concentration. The intra-assay precision and accuracy were calculated using replicate determinations (*n* = 6) for each concentration of the flavonoids and the biotransformation products that were added to the plasma samples during a single analytical run. The inter-assay precision and accuracy were calculated using replicate determinations (*n* = 6) for each concentration of the flavonoids and the biotransformation products, and these were made on three separate days. Accuracy was calculated using the following equation: (% Bias) = [(Cobs–Cnom)/Cnom] × 100. The precision was calculated from the observed concentrations as follows: RSD = [standard deviation (SD)/Cobs] × 100. Accuracy (Bias) and precision (RSD) values were within ±15%, covering a range of actual experimental concentrations that were considered acceptable.

### 3.14. Recovery (Extraction Efficiency)

The extraction efficiency of the flavonoids and the biotransformation products was determined by analyzing a series of replicates (*n* = 6) of quality control (QC) samples; 0.8, 4.0, and 10 μg/mL for mice plasma, representing low, medium, and high QCs, respectively. Recovery was calculated by comparing the peak areas of the flavonoids and the biotransformation products that were added into the blank samples and extracted using the protein precipitation procedure with those obtained from the flavonoids, and the biotransformation products spiked/jumped directly into post-protein precipitation solvent at three QC concentration levels.

### 3.15. Stability Study

The stability of the flavonoids and the biotransformation products in mice plasma was assessed by analyzing the replicates (*n* = 6) of the QC samples at three different concentrations (0.8, 4.0, and 10 μg/mL) in mice plasma. The investigation enclosed here expected manipulation conditions during all the sample storage and process periods, which included the stability data from the freeze/thaw, bench-top, autosampler, and long–term stability tests. 

For all stability studies, fresh QC samples were evaluated by using a freshly prepared standard curve for the measurements. The concentrations obtained from all the stability studies were compared with the fresh QC samples, and the percentage concentration deviation was calculated. The analytes were considered stable in the mouse plasma when the concentration difference between the freshly prepared samples and the stability samples was less than 15%.

### 3.16. Pharmacokinetic Analysis

To evaluate the suitability of the assay for the pharmacokinetic studies, 200 mg/kg of the active fraction was orally administered to the animals. Ten animals were used in each group at different times (0, 5, 10, 15, 30, 60, 120, 240, 480, 720, and 1440 min). Pharmacokinetic calculations were performed using the observed data. All data was subsequently processed using the PKSolver add-in program for Microsoft Excel written in Visual Basic for Applications. All values obtained were expressed in mean ± standard deviation.

For the pharmacokinetic assay, the selection was made either from a one-compartment or a two-compartment model, with the use of both the Akaike information criterion (AIC) and the Schwarz criterion (SC). Using these two criteria, one can select which model is more suited to being adjusted, to the point where it reaches the lowest values of the AIC or SC criteria, which means that the chosen model is more parsimonious (less parameters required) and best fits the data (low error prediction).

## 4. Conclusions

Flavonoids with potential anxiolytic activity are pro-drugs. The active compounds are *m*-HPAA, *p*-HPAA, and DOPAC, which are derivatives of the biotransformation of the aglycones kaempferol and quercetin.

## Figures and Tables

**Table 1:** Stability profile of anxiolytic *T. americana* flavonoid fractions or the flavonoid biotransformation products found in mouse plasma (matrix) under different handling conditions, (*n* = 6).

	Compound	Nominal Conc. (µg/mL)	Observed Conc. (µg/mL)				Accuracy Bias (%)				RSD (%)		
			0 h		8 h	24 h	0 h		8 h	24 h	0 h	8 h	24 h
Room temperature (25 °C)	Rutin	1.75	1.74 ± 0.11		1.65 ± 0.10	1.64 ± 0.10	5.76		5.46	0.33	6.74	6.58	6.57
		3.50	3.39 ± 0.16		3.67 ± 0.30	3.38 ± 0.27	3.17		−4.97	2.93	4.76	8.25	8.14
		7.00	7.27 ± 0.41		7.17 ± 0.21	7.13 ± 0.24	−1.99		−2.45	−3.93	5.72	2.95	3.39
	Quercetin glucoside	11.02	11.16 ± 0.27		10.84 ± 0.67	11.35 ± 0.61	−1.24		1.62	−2.98	2.42	6.18	5.38
		22.18	20.86 ± 1.35		20.84 ± 1.24	20.44 ± 1.65	5.94		6.02	7.83	6.46	5.99	8.08
		44.37	43.10 ± 2.20		42.48 ± 1.06	41.37 ± 1.17	2.86		4.28	6.76	5.10	2.49	2.84
	Quercitrin	11.02	11.27 ± 0.75		11.56 ± 0.78	10.99 ± 0.72	−2.26		−4.83	0.30	6.67	6.74	6.59
		22.18	22.94 ± 1.87		22.65 ± 1.85	23.11 ± 1.89	−3.39		−2.11	−4.15	8.17	8.16	8.18
		44.37	44.12 ± 2.82		44.96 ± 1.84	43.90 ± 1.61	0.56		−1.33	1.05	6.40	4.10	3.66
	Tiliroside	11.02	10.54 ± 0.74		10.33 ± 0.61	10.53 ± 0.79	4.59		6.26	4.48	7.09	5.89	7.50
		22.18	21.44 ± 2.13		22.84 ± 2.57	20.60 ± 2.05	3.36		−2.95	7.14	9.98	9.95	9.99
		44.37	43.77 ± 4.28		44.65 ± 4.37	41.25 ± 2.04	1.35		−0.64	7.03	9.80	9.79	9.80
	Kaempferol	1.12	1.16 ± 0.099		1.21 ± 0.83	1.15 ± 0.098	−3.29		−7.28	−2.28	8.53	6.92	8.52
		2.24	2.24 ± 0.20		2.15 ± 0.19	2.42 ± 0.22	0.53		4.60	−8.12	9.06	9.03	9.10
		4.50	4.45 ± 0.41		4.32 ± 0.21	4,.17 ± 0.34	−4.64		−1.74	1.77	9.34	5.05	8.26
	*m*-HPAA	1.95	1.91 ± 0.2		1.91 ± 0.03	1.90 ± 0.04	0.47		2.23	2.73	0.33	1.61	1.98
		7.81	7.80 ± 0.01		7.98 ± 0.12	7.82 ± 0.01	0.13		2.18	0.09	0.09	1.51	0.06
		31.25	31.40 ± 0.10		31.53 ± 0.19	31.34 ± 0.07	1.88		0.90	0.30	1.35	0.63	0.22
	*p*-HPAA	1.95	1.92 ± 0.02		1.92 ± 0.02	1.93 ± 0.01	1.44		1.40	0.85	1.03	1.01	0.60
		7.81	7.86 ± 0.11		7.94 ± 0.11	7.89 ± 0.05	0.65		2.05	1.01	0.46	1.42	0.71
		31.25	31.76 ± 0.28		31.64 ± 0.28	31.64 ± 0.31	1.64		1.26	1.26	1.14	0.88	0.88
	DOPAC	1.95	1.98 ± 0.020		1.92 ± 0.02	1.91 ± 0.03	0.02		0.03	0.03	1.26	1.46	1.61
		7.81	7.85 ± 0.032		7.86 ± 0.03	7.78 ± 0.02	0.03		0.03	0.02	0.40	0.41	0.27
		31.25	31.61 ± 0.26		31.64 ± 0.24	31.53 ± 0.19	0.26		0.25	0.19	0.82	0.80	0.62
		**Nominal Conc. (µg/mL)**	**Observed Conc. (µg/mL)**				**Accuracy Bias (%)**				**RSD (%)**		
			**Autosampler (4 °C; 8 h)**	**Long-term (−70 °C; 1 month)**			**Autosampler (4 °C; 8 h)**	**Long-term (−70°C; 1 month)**			**Autosampler (4°C; 8 h)**	**Long-term (−70°C; 1 month)**	
Storage stability	Rutin	1.75	1.86 ± 0.19	1.69 ± 0.12			−6.41	2.92			10.58	7.24	
		3.50	3.44 ± 0.15	3.51 ± 0.28			1.58	−0.45			4.39	7.97	
		7.00	7.08 ± 0.34	7.10 ± 0.38			−1.17	−1.56			4.81	5.43	
	Quercetin glucoside	11.02	11.22 ± 0.59	11.41 ± 0.71			2.40	6.22			5.29	6.25	
		22.18	20.96 ± 1.31	20.57 ± 2.06			5.48	7.26			6.28	10.02	
		44.37	43.30 ± 2.02	41.60 ± 2.26			−1.82	−3.52			4.66	5.45	
	Quercitrin	11.02	11.62 ± 1.02	11.64 ± 0.99			−5.47	−2.89			8.82	8.76	
		22.18	22.79 ± 2.26	22.49 ± 1.66			−2.73	−1.41			9.92	7.40	
		44.37	44.44 ± 4.52	44.17 ± 2.95			−0.17	0.44			10.17	6.69	
	Tiliroside	11.02	10.58 ± 0.81	10.40 ± 0.99			3.97	5.54			7.69	9.54	
		22.18	22.94 ± 2.25	21.41 ± 1.82			−3.45	3.42			9.81	8.51	
		44.37	42.74 ± 3.40	44.26 ± 2.49			3.66	0.24			7.97	5.64	
	Kaempferol	1.12	1.21 ± 0.07	1.15 ± 0.10			−7.77	−2.77			6.56	8.67	
		2.24	2.24 ± 0.20	2.15 ± 0.19			0.50	4.13			9.09	9.07	
		4.50	4.19 ± 0.37	4.35 ± 0.39			1.24	−2.47			8.95	9.03	
	*m*-HPAA	1.95	1.93 ± 0.04	1.92 ± 0.03			0.09	0.90			0.22	0.63	
		7.81	7.82 ± 0.01	7.80 ± 0.12			0.30	2.18			0.06	1.51	
		31.25	31.34 ± 0.07	31.20 ± 0.19			2.73	2.23			1.98	1.61	
	*p*-HPAA	1.95	1.93 ± 0.02	1.92 ± 0.01			1.26	1.26			0.88	0.88	
		7.81	7.89 ± 0.11	7.97 ± 0.05			2.05	1.01			1.42	0.71	
		31.25	31.64 ± 0.28	31.64 ± 0.28			1.40	0.85			1.01	0.60	
	DOPAC	1.95	1.91 ± 0.03	1.99 ± 0.03			0.88	1.15			0.80	0.62	
		7.81	7.78 ± 0.02	7.85 ± 0.03			0.38	0.58			0.41	0.27	
		31.25	31.61 ± 0.19	31.53 ± 0.25			2.23	2.11			1.46	1.61	

**Table 2:** Variation in plasma concentration of the flavonoid biotransformation products in the first 24 h after the oral administration of a single dose of 200 mg/kg of the anxiolytic fraction.

Plasma Concentration (μg/mL)								
Time (min)	Rutin	Quercetin Glucoside	Quercitrin	Tiliroside	Kaempferol	*m*-HPAA	*p*-HPAA	DOPAC
5	ND	ND	ND	ND	ND	4.67 ± 0.27	5.40 ± 0.36	6.60 ± 0.43
10						5.06 ± 0.22	6.71 ± 0.44	6.43 ± 0.35
15						4.75 ± 0.29	6.01 ± 0.33	7.73 ± 0.61
30						4.52 ± 0.28	5.38 ± 0.51	7.17 ± 0.52
60						4.29 ± 0.13	5.31 ± 0.54	6.88 ± 0.43
120						4.17 ± 0.33	5.29 ± 0.32	6.34 ± 0.41
240						4.11 ± 0.23	5.15 ± 0.23	6.26 ± 0.32
480						4.04 ± 0.25	5.08 ± 0.24	6.10 ± 0.34
720						3.94 ± 0.19	4.99 ± 0.35	6.01 ± 0.54
1440						3.79 ± 0.28	4.91 ± 0.21	5.93 ± 0.49

ND. Not detected.

**Table 3:** Pharmacokinetic parameters of the flavonoid biotransformation products present in the anxiolytic fraction of *T. americana* in plasma of ICR mice; *n* = 10. The analytes fit into the two-compartment model.

	*m*-HPAA	*p*-HPAA	DOPAC	Units.
A	3.49	3.19	1.23	µg/mL
B	3.36	3.75	6.16	µg/mL
k_a_	0.24	0.36	0.43	l/min
k_10_	0.0009	0.0006	3.18 × 10^−5^	1/min
k_12_	0.009	0.007	0.002	1/min
k_21_	0.01	0.009	0.0096	1/min
t_1/2_α	35.08	42.25	59.95	min
t_1/2_β	1501.88	1993.85	26,012.93	min
t_1/2_ka	2.84	1.92	1.61	min
α	0.02	0.02	0.01	1/min
β	0.0005	0.0003	2.66 × 10^−5^	1/min
V/F	30.45	29.46	27.21	(mg/Kg)/(µg/mL)
CL/F	0.026	0.018	0.0008	(mg/Kg)/(μg/mL)/min
T_max_	14.10	11.16	12.90	min
C_max_	5.76	6.26	7.18	µg/mL
AUC_0→∞_	7434.23	10,962.77	231,124.48	µg/mL min
AUC_0→1440_	3685.97	4423.64	8786.10	µg/mL min
MRT	2124.58	2831.66	37,514.31	min
Diagnostics				
SS	0.78	0.39	0.89	
R^2^	0.99	0.99	0.998	
AIC	7.49	0.77	8.84	
SC	9.00	2.27	10.36	

The data was analyzed according to the following equations: (one compartment model) C_p_ = A**e**^-kdt^ + C**e**^-kat^ and (two-compartment model) C_p_ = A**e**^-αt^ + B**e**^-βt^ + C**e**^-kdt^. C_p_ is the plasma concentration (μg/mL); k_d_, β, are disposition rate constants (min^−1^); ka is the absortion rate constant (min^−1^); A, B, and C are the coefficients (μg/mL). The parameters shown in Table 3 A and B are for *m-*HPAA, *p-*HPAA, and DOPAC plasma concentration in the central and peripheral compartments, respectively; k_a_ is apparent first-order absorption rate constant; k_10_ is the apparent first-order elimination rate constant from the central compartment; k_12_ is the apparent first-order transfer rate constant from the central compartment to the peripheral compartment; k_21_ is the apparent first-order transfer rate constant from the peripheral compartment to the central compartment; t_1/2_α is the absorption half time for the central compartment; t_1/2_β is the absorption half time for the peripheral compartment; t_1/2_ka is the absorption half time; α and β are the empirical constants corresponding to the coefficients of the exponents of the values of A and B; V/F is the apparent volume of distribution; CL/F is the apparent clearance; T_max_ is the time to maximal concentration; C_max_ is the maximal concentration; AUC_0→∞_ and AUC_0–1440 min_ are the areas under the plasma curve from 0 to infinity and from 0 to 1440 min, respectively; and MRT is the mean residence time. The goodness of fit were assessed with Sum of Squares (SS), Akaike Information Criteria (AIC), Schwarz Criteria (SC), and a determination coefficient (R^2^).

**Table 4:** Concentrations used in the different tests for the validation of the analytical methods.

Compound	Concentration (µg/mL)	Level
Rutin	1.75	Low
	3.50	Medium
	7.00	High
Quercetin glucoside	11.02	Low
Quercitrin	22.18	Medium
Tiliroside	34.37	High
Kaempferol	1.125	Low
	2.25	Medium
	4.50	High
*m-*HPAA	1.95	Low
*p-*HPAA	7.81	Medium
DOPAC	31.25	High

## Supplementary Materials

The following are available online. Table S1: Calculation of limit of detection (LOD) and limit of quantification (LOQ) values; Table S2: Quantification of the flavonoid fraction of anxiolytic *T. americana* or the biotransformation products from flavonoids in mouse plasma (matrix) and the determination of precision and accuracy. Table S3: Recovery yield of the flavonoid fraction of anxiolytic *T. americana* or biotrans-formation products from flavonoids in mouse plasma (matrix), (*n* = 6).
