# Supplementary material for: Pharmacokinetic Study of Biotransformation Products from an Anxiolytic Fraction of Tilia americana
Source: Molecules. 2017 Jul 27;22(8):1260. doi: 10.3390/molecules22081260 (PMC6152131; doi:10.3390/molecules22081260)
Supplement: Supplementary file 1 [file molecules-22-01260-s001.pdf]

Table S1. Calculation of limit of detection (LOD) and limit of quantification (LOQ) values.

Compound	The slope of the calibration curve (S)	The standard deviation (σ)	Limit of detection (LOD)= 3.3σ/S	Limit of quantitation (LOQ)=10σ/S
Rutin	0,0917	0,0048601	0,1749	0,53
Quercetin glucoside	0,0117	0,0045045	1,2705	3,85
Quercitrin	0,0207	0,0079488	1,2672	3,84
Tiliroside	0,0744	0,0280488	1,2441	3,77
Kaempferol	0,0091	0,00078624	0,28512	0,864
mHPAA	0,091	0,0018018	0,06534	0,198
pHPAA	0,073	0,0031755	0,14355	0,435
DOPAC	0,104	0,0015392	0,04884	0,148

Table S2. Quantification of the flavonoid fraction of anxiolytic T. americana or the biotransformation products from flavonoids in mouse plasma (matrix) and the determination of precision and accuracy

Compound	Nominal Concentration (μg/mL)	Observed Concentration (μg/mL) ±S.D.	Accuracy (% Bias)	RDS (%)
Rutin Intra-day	1.75	1.70±0.05	2.39	2.82
	3.50	3.39±0.07	3.09	2.08
	7.00	6.79±0.26	2.89	3.80
Rutin Inter-day	1.75	1.75±.056	-0.04	3.23
	3.50	3.41±0.14	2.47	3.98
	7.00	7.16±0.42	-2.39	5.80
Quercetin glucoside Intra-day	11.02	10.45±0.56	5.16	5.41
	22.18	21.41±0.45	3.46	2.11
	44.37	43.83±1.02	1.21	2.34
Quercetin glucoside Inter-day	11.02	10.32±0.32	3.60	3.06
	22.18	21.81±0.37	1.69	1.72
	44.37	44.15±0.45	0.47	1.03
Quercitrin Intra-day	11.02	10.81±0.16	1.91	0.15
	22.18	21.46±0.18	3.25	0.84
	44.37	44.24±0.94	0.29	2.13
Quercitrin Inter-day	11.02	10.79±0.49	2.06	0.46
	22.18	21.59±0.57	2.64	0.26
	44.37	45.25±0.38	-1.98	0.86
Tiliroside Intra-day	11.02	10.80±0.30	2.00	2.75
	22.18	22.80±0.73	-2.78	3.19
	44.37	43.47±1.85	2.01	4.25
Tiliroside Inter-day	11.02	11.05±0.25	-0.19	2.27
	22.18	21.37±0.90	3.68	4.21
	44.37	43.69±0.82	1.51	1.89

Kaempferol Intra-day	1.12	1.05±0.04	-3.95	4.25
	2.24	2.23±0.11	0.59	5.09
	4.50	4.47±0.05	0.55	1.18
Kaempferol Intra-day	1.12	1.07±0.05	-5.46	5.47
	2.24	2.37±0.05	-5.68	2.32
	4.50	4.34±0.17	3.43	3.92
m-HPAA Intra-day	1.95	2.05±0.07	4.97	0.70
	7.81	8.00±0.13	2.44	1.68
	31.25	31.56±0.22	0.99	3.35
m-HPAA Inter-day	1.95	2.05±0.07	0.95	0.70
	7.81	8.00±0.13	2.47	1.70
	31.25	31.55±0.22	4.91	3.31
p-HPAA Intra-day	1.95	1.990±02	0.51	0.36
	7.81	7.90±0.06	1.19	0.83
	31.25	31.41±0.11	2.05	1.42
p-HPAA Inter-day	1.95	1.99±0.06	2.27	1.57
	7.81	7.90±0.06	1.23	0.83
	31.25	31.37±0.08	0.38	0.27
DOPAC Intra-day	1.95	2.06±0.08	5.65	3.78
	7.81	7.92±0.07	1.36	0.95
	31.25	31.52±0.19	0.88	0.61
DOPAC Inter-day	1.95	2.06±0.07	5.65	3.78
	7.81	7.92±0.07	1.36	0.95
	31.25	31.52±0.19	0.88	0.61

The intra-day and inter-day precision (% RSD) in mouse plasma was <15%. The recovery was approximately 85% to 98% in the plasma. (n=6)

Table S3. Recovery yield of the flavonoid fraction of anxiolytic T. americana or the biotransformation products from flavonoids in mouse plasma (matrix), (n = 6).

Compound	Spiked Concentration (μg/mL)	Observed Concentration (μg/mL) ± S.D.	Recovery Index ± S.D.	RSD (%)
Rutine	1.75	1.66±0.13	0.95±0.07	7.38
	3.50	3.30±0.18	0.94±0.05	5.39
	7.00	6.82±0.40	0.97±0.06	5.91
Quercetin glucoside	11.02	10.79±0.20	0.95±0.06	6.67
	22.18	21.60±0.60	0.97±0.03	2.80
	44.37	43.05±2.45	0.95±0.04	4.55
Quercitrin	11.02	11.59±0.98	1.05±0.09	8.48
	22.18	21.80±1.95	0.98±0.08	8.95
	44.37	43.48±1.11	0.98±0.02	2.57

Tiliroside	11.02	11.04±0.83	1.00±0.07	7.55
	22.18	21.39±1.42	0.96±0.06	6.67
	44.37	43.42±2.33	0.98±0.05	5.37
Kaempferol	1.125	1.05±0.04	0.94±0.08	3.85
	2.25	2.02±0.14	0.90±0.06	7.22
	4.50	4.03±0.36	0.94±0.04	9.03
m-HPAA	1.95	1.91±0.10	0.98±0.08	2.64
	7.81	7.80±0.01	0.99±0.02	2.04
	31.25	31.14±0.10	0.99±0.03	3.01
p-HPAA	1.95	1.90±0.03	0.97±0.04	4.15
	7.81	7.80±0.02	0.98±0.02	2.63
	31.25	31.23±0.02	0.99±0.03	2.80
DOPAC	1.95	1.90±0.13	0.98±0.02	2.78
	7.81	7.80±0.03	0.99±0.02	2.91
	31.25	31.23±0.03	0.99±0.03	3.14
